# Women With Congenital Adrenal Hyperplasia Have Favorable Pregnancy Outcomes but Prolonged Time to Conceive

**DOI:** 10.1210/jendso/bvae211

**Published:** 2024-12-06

**Authors:** Matthias K Auer, Clara E Minea, Marcus Quinkler, Irina Bancos, Felix Beuschlein, Gesine Meyer, Christian Lottspeich, Martin Bidlingmaier, Eva Rieger, Hanna F Nowotny, Lea Tschaidse, Henrik Falhammar, Rosario Pivonello, Chiara Simeoli, Nicole Reisch

**Affiliations:** Medizinische Klinik and Poliklinik IV, Klinikum der Universität München, LMU München, Munich 80336, Germany; Medizinische Klinik and Poliklinik IV, Klinikum der Universität München, LMU München, Munich 80336, Germany; Endocrinology in Charlottenburg, Berlin 10627, Germany; Division of Endocrinology, Metabolism, Diabetes and Nutrition, Mayo Clinic, Rochester, MN 55905, USA; Universitätsspital Zürich (USZ), Klinik für Endokrinologie, Diabetologie und Klinische Ernährung, Universitätsspital Zürich (USZ) und Universität Zürich (UZH), Zürich 8091, Switzerland; Medical Clinic 1, Division of Endocrinology, Diabetes and Nutrition, University Hospital, Goethe University Frankfurt, Frankfurt 60590, Germany; Medizinische Klinik and Poliklinik IV, Klinikum der Universität München, LMU München, Munich 80336, Germany; Medizinische Klinik and Poliklinik IV, Klinikum der Universität München, LMU München, Munich 80336, Germany; Medizinische Klinik and Poliklinik IV, Klinikum der Universität München, LMU München, Munich 80336, Germany; Medizinische Klinik and Poliklinik IV, Klinikum der Universität München, LMU München, Munich 80336, Germany; Medizinische Klinik and Poliklinik IV, Klinikum der Universität München, LMU München, Munich 80336, Germany; Department of Endocrinology, Karolinska University Hospital, Stockholm 171 76, Sweden; Department of Molecular Medicine and Surgery, Karolinska Institutet, Stockholm 171 76, Sweden; Dipartimento di Medicina Clinica e Chirurgia, Sezione di Endocrinologia, Università Federico II di Napoli, Naples 80131, Italy; Dipartimento di Medicina Clinica e Chirurgia, Sezione di Endocrinologia, Università Federico II di Napoli, Naples 80131, Italy; Medizinische Klinik and Poliklinik IV, Klinikum der Universität München, LMU München, Munich 80336, Germany

**Keywords:** CAH, congenital adrenal hyperplasia, classic, nonclassic, pregnancy, outcomes

## Abstract

**Objective:**

To study pregnancy outcomes and complications in women with congenital adrenal hyperplasia (CAH).

**Methods:**

A retrospective multicenter study was conducted at tertiary reference centers in 5 countries (Austria, Germany, Italy, Sweden, USA), including 72 adult women with CAH (nonclassic [NC] n = 34, simple virilizing [SV] n = 21, salt wasting [SW] n = 17).

**Results:**

A total of 133 pregnancies, 112 live births, and 25 abortions were documented. Prolonged latency to pregnancy was observed (median 11 months in SW, 24 months in SV, 8 months in NC), with a notable use of fertility-enhancing medication (25.6%) and assisted reproductive techniques (30.8%). Over half of the women in each group took more than 12 months to conceive. The average number of live births (1.4-1.6 children per woman) was similar across CAH phenotypes and comparable to the general population. Spontaneous abortion rates (18.0%) were also similar across phenotypes. Primary cesarean section rates (60.9%) were higher than in the general population, though 23.8% of women with SV and 29.4% of women with SW gave birth naturally, despite most having undergone genital surgery. Children categorized as small for gestational age were 20.5%. Pregnancy, delivery, and postpartum complications were rare for mothers and neonates.

**Conclusion:**

The study indicates a prolonged latency to pregnancy and high use of fertility treatments in CAH patients, regardless of phenotype. Abortion rates were not increased, and overall pregnancy and perinatal outcomes were favorable.

Congenital adrenal hyperplasia (CAH) arises due to mutations affecting critical enzymes responsible for steroid hormone synthesis. The gene encoding 21-alpha-hydroxylase (21OH) is the most frequently impacted. Under normal physiological conditions, 21OH facilitates steps in the synthesis of both glucocorticoids and mineralocorticoids. Variants in the context of CAH are associated with different degrees of loss of enzymatic activity, resulting in a deficit of these vital hormones. Glucocorticoid deficiency prompts the excessive secretion of pituitary adrenocorticotropic hormone (ACTH) and hence stimulation of the adrenal cortex, culminating in the overproduction of adrenal androgens [1, 2]. The manifestations of CAH can vary based on the severity of the enzymatic malfunction: these relate to 3 notable phenotypes: the nonclassic CAH (NC), which represents the mildest form, characterized by hyperandrogenism and often menstrual disturbances in women; the simple virilizing (SV) CAH, marked by both hyperandrogenism and a glucocorticoid deficiency; and lastly, the salt wasting (SW) CAH which, being the most severe form, presents with an additional severe mineralocorticoid deficiency [3].

Excessive adrenal steroid hormones can disrupt the function of the hypothalamus-pituitary-gonadal axis and compromise endometrial integrity [2]. As a consequence, women with CAH may face challenges such as reduced fertility, diminished reproduction rates, and adverse pregnancy outcomes [4-6]. Beyond the hormonal imbalance, urogenital anomalies due to genital virilization and subsequent need for genital surgery may affect pregnancy outcomes or preclude vaginal intercourse in the first place. Dissatisfaction with sexual life due to surgical complications may be a persistent problem resulting in reduced sexual confidence [7-9]. Prior studies have shown that the low pregnancy rates reported earlier among women with CAH are primarily due to inadequate treatment, as well as the fact that fewer women in this group seek motherhood, and are not due to a general inability to conceive [10].

With regard to complicated pregnancy courses and adverse outcomes, there are reports on higher rates of gestational diabetes and of children born small for gestational age (SGA) [11, 12]. However, the limited scope of some studies, whether due to small sample sizes or reliance on epidemiological samples, often hampers a comprehensive understanding of the factors influencing these outcomes. Our study endeavors to delineate the pregnancy trajectories of all 3 phenotypes of CAH in a multicentric cohort and to identify potentially modifiable factors of adverse pregnancy outcomes.

## Methods

### Subjects

We conducted a multicenter retrospective cohort study including data from centers in Berlin, Frankfurt, Munich (all Germany), Naples (Italy), Rochester (USA) and Stockholm (Sweden). Furthermore, patients from the Austrian patient support group “Netzwerk AGS Österreich” were included (Supplement Table S1 [13]). With the exception of one patient from the center in Munich, who opted out for personal reasons, all women who were approached on the subject were willing to participate. The final study group comprised 72 women with CAH (34 NC; 21 SV; 17 SW) who were pregnant at least once in their life until study inclusion. For those who had multiple children, detailed histories of up to 3 pregnancies were documented. The study protocol had been approved by the local ethics committees and all participants provided their written informed consent.

### Questionnaires

For data collection self-designed questionnaires were used. They included questions about conception, pregnancy course, and outcome. Two different questionnaires were implemented in the study: one was filled out by the patients themselves and the other by their endocrinologists to complete the information. Disease-specific data were verified by the treating physician using patients' medical files when available. In addition, patients were asked to provide copies of their maternity records. Missing data are reported. If not stated otherwise, reported percentages are referring to those women with complete data.

### Definitions

#### Genotypes

Patients were assigned to genotype groups based on the in vitro 21-hydroxylase enzyme activity reported in the literature and the predicted functional consequences for the phenotype. Generally, the phenotype is determined by the allele carrying the less severe mutation in CAH [14]. The classification is as follows: *0* (null) indicates no enzymatic activity; *A* refers to 0% to 1% enzymatic activity (eg, intron 2 splice site mutations); *B* indicates 1% to 2% residual in vitro enzyme activity; and *C* includes mutations such as p.Pro30Leu, p.Val281Leu, and p.Pro453Ser, with activity greater than 20% to 30%. While the genotype-phenotype correlation is generally strong for the *0* (null) and *C* groups, there is significant variability within mutation groups of intermediate severity.

#### Apgar score

To standardize the adaptation response of mature newborns, the Apgar score (a 5-factor assessment based on heart rate, breathing, muscle tone, reflex irritability, and color, also referred to as appearance, pulse, grimace, activity, and respiration) was used [15]. A 5-minute score of 10 to 7 points was defined as “good adaptation,” a score of 6 to 4 points as “slightly limited adaptation,” and a score of less than 4 points as “severely limited adaptation.”

#### Gestational age and intrauterine growth

Based on gestational age post menstruation, births were classified into preterm (up to 36 + 6), term (between 37 + 0 and 41 + 6), and post-term (from 42 + 0) [16-18]. Intrauterine growth was assessed using the growth curves of Voigt et al [19]. Neonates whose birth weight was below the 10th percentile of children with the same age and sex were defined as small for gestational age (SGA). Conversely, newborns whose birth weight was above the 90th percentile of children with the same age and sex were designated large for gestational age (LGA) [20, 21].

#### Glucocorticoid equivalents

The hydrocortisone equivalent doses of glucocorticoid (HCeq) under different regimens were calculated using the following factors: hydrocortisone = 1, prednisone/prednisolone = 4, dexamethasone = 80 [22].

### Statistical Analysis

Statistical analysis was performed using IBM SPSS Statistics Version 28.0.1.0. For nominally and ordinally scaled test variables, either the Chi-squared test or Fisher exact test was used for group comparison. For metric variables, the Kruskal-Wallis with post hoc Dunn-Bonferroni test and the Wilcoxon signed-rank test were used as appropriate. To explore potential influential factors of birthweight, a multivariate analysis was conducted using a linear mixed model including week of birth and sex, HCeq and type of glucocorticoid (GC) used during pregnancy and phenotype as fixed factors. A two-sided *P* value of <.05 was considered statistically significant.

## Results

### General Characteristics of the Cohort

The median age at the time of evaluation did not differ between the groups (*P* = .173). Women with classic CAH were diagnosed in early childhood, whereas the average age at diagnosis for women with NC CAH was 21.1 years (*P* < .001). Genetic information was available from 47.0% of patients in the NC CAH group, 42.8% in the SV group, and 52.9% in the SW group. From those women for whom genetic information was available, 100% belonged to genotype group C in the NC CAH group, and 100% of those with an SV phenotype had a B genotype. The SW group was more diverse, with 1 woman (12.5%) having a “null” genotype, while 62.5% belonged to group A and 25% to group B.

Six women (21.4%) with NC CAH were diagnosed after their first pregnancy. All women with SW and 70.0% of women with SV underwent corrective surgery for the genitourinary tract. In the NC group, 68.7% had used oral contraceptives before childbearing, compared to 64.7% in the SV group and 46.7% in the SW group (not significant [n.s.]). The median duration of hormonal contraceptive use was 9 years across the entire cohort (n.s.) (Table 1). At the time of data collection, 3 children had not yet been born.

**Table 1.** Overview of variables before conception

	NC (N = 34)	SV (N = 21)	SW (N = 17)	*P*	NC vs SV	NC vs SW	SV vs SW
	Median (IQR)	Median (IQR)	Median (IQR)				
Age at evaluation, years	40 (24.25-48.25)	46 (39-62)	43.5 (33.75-52.75)	.173	—	—	—
First genital surgery, years	—	6 (4.6-11.8)	3 (2-7)	.070	—	—	—
Second genital surgery, years	—	14 (5.0-23)	11 (7-13)	.739	—	—	—
Age menarche, years	12 (11-13)	13 (12-14)	13 (12-14)	.112	—	—	—
	**N (%)**	**N (%)**	**N (%)**				
No genital surgery	(100)	4 (23.5)	0 (0)	**<.001**	**<.001**	**<0.001**	0.153
One urogenital surgery	—	11 (64.7)	10 (62.5)				
Two urogenital surgeries	—	2 (11.8)	4 (25.0)				
More than two	—	0	2 (12.5)				
Missing	—	4 (19.0)	1 (5.9)				

	Median (IQR)	Median (IQR)	Median (IQR)				
Duration of oral contraceptive use before first pregnancy attempt, months	9 (6-14)	4.5 (3-15)	9 (8-11)	.188	—	—	—
	**N (%)**	**N (%)**	**N (%)**				
Rate of oral contraceptive intake before pregnancy, n (%)	22 (68.7)	11 (64.7)	7 (46.7)	.337	—	—	—
Rate of urogenital surgery, n (%)	—	14 (70.0)	16 (100.0)	.**016**	—	—	**0.016**
Diagnosis after first pregnancy, n (%)	6 (21.4)	0 (0)	0 (0)	.**018**	**0.037**	**0.043**	—
Genotype							
0	0 (0)	0 (0)	1 (12.5)	**<.001**	**<0.001**	**<0.001**	**<0.001**
A	0 (0)	0 (0)	5 (62.5)				
B	0 (0)	12 (100)	2 (25.0)				
C	18 (100)	0 (0)	0 (0)				
Missing	16 (47.0)	9 (42.8)	9 (52.9)				

χ²-Test/Fishers exact test, Kruskal-Wallis-Test with post hoc Dunn-Bonferroni-Test. Bold numbers indicate significant group differences. Percentages refer to those women, information was available. Missing information is reported separately.

Abbreviations: IQR, interquartile range; NC, nonclassic; SV, simple virilizing; SW, salt wasting.

### Comorbidities

In our cohort, hypothyroidism was the most common comorbidity (N = 16). Among these, 11 were receiving levothyroxine replacement therapy. Hypothyroidism was significantly more common in women with NC CAH than in women with SW (*P* = .023). Notably, there were also 3 patients with arterial hypertension and 3 diagnosed with diabetes mellitus (2 with type 2 and 1 with type 1 diabetes) (see Supplement Table S2 [13]).

None of the mothers had drug abuse or were smoking when trying to conceive or during pregnancy. Fathers did not have any known health conditions affecting fertility.

### Pregnancies

On average, each woman in the study had given birth to 1.6 children (NC CAH: 1.6; SV: 1.6; SW: 1.4 [n.s.]). In the total cohort, there were 133 documented pregnancies. Of these pregnancies, 112 resulted in live births, 24 ended in spontaneous abortion, and 1 in a therapeutic abortion. Among the 133 pregnancies, 7 were twins. The number of pregnancies, and respective children born, did not differ between groups (*P* = .683). The sex of the child was reported in 99 pregnancies, with 50 being male and 49 being female. The 3 phenotype groups did not show any significant difference in the number of live births, spontaneous abortions, or therapeutic abortions (Table 2).

**Table 2.** Pregnancies

		NC (N = 34) Median (IQR)	SV (N = 21) Median (IQR)	SW (N = 17) Median (IQR)				
					*P*	NC vs SW	NC vs SV	SV vs SW
Average live births		2 (1-2)	1 (1-2)	1 (1-2)	.315	—	—	—
		**N (%)**	**N (%)**	**N (%)**				
Pregnancies (N = 133)	Total	62	34	37	.772	—	—	—
	1	14 (41.2)	12 (57.1)	5 (29.4)				
	2	15 (44.1)	6 (28.6)	7 (41.2)				
	3	3 (8.8)	2 (9.5)	3 (17.6)				
	4	1 (2.9)	1 (4.8)	1 (5.9)				
	5	1 (2.9)	0 (.0)	1 (5.9)				
Live births (N = 112)	Total	55	30	27	.683	—	—	—
	0	2 (5.9)	0 (.0)	0 (.0)				
	1	14 (41.2)	13 (61.9)	9 (52.9)				
	2	15 (44.1)	7 (33.3)	6 (35.3)				
	3	2 (5.9)	1 (4.8)	2 (11.8)				
	5	1 (2.9)	0 (0.0)	0 (0.0)				
Spontaneous abortions (N = 24)	Total	9	5	10	0.360	—	—	—
	0	28 (82.4)	16 (76.2)	11 (64.7)				
	1	4 (11.8)	5 (23.8)	4 (23.5)				
	2	1 (2.9)	0 (0.0)	1 (5.9)				
	3	1 (2.9)	0 (0.0)	0 (0.0)				
	4	0 (0.0)	0 (0.0)	1 (5.9)				
Therapeutic abortions (N = 1)		0 (0.0)	1 (4.8)	0 (0.0)	.297	—	—	—
Total women using ART or fertility-enhancing medication at any pregnancy	Naturally	17 (58.6)	10 (55.6)	12 (85.7)	.169	—	—	—
	Fertility-enhancing medication	8 (27.6)	1 (5.6)^#^	1 (7.6)	.105	—	—	—
	ART	4 (13.8)	7 (38.9)	1 (7.1)	**.05**	**0.05**	0.663	**0.046**
	Missing	5 (14.7)	3 (14.3)	3 (17.6)				
Conception first pregnancy	Naturally	18 (66.7)	9 (58.8)	9 (90.0)	.232	—	—	—
	Fertility-enhancing medication	7 (25.9)	2 (12.5)^#^	0 (0.0)	.168	—	—	—
	ART	2 (7.4)	5 (31.3)	1 (10)	**.049**	**0.05**	0.068	**0.046**
	Missing (N)	7 (20.6)	5 (23.8)	7 (41.2)				

		Median (IQR)	Median (IQR)	Median (IQR)				
Age first pregnancy, years	30 (27-34)	33 (26-35)	29.5 (27-33)	0.134				
Age second pregnancy, years	32 (29.8-35)	33 (29-36)	33 (32-35)	0.527	—	—	—	—
Age third pregnancy, years	38 (34-39)	31 (31-31)	35 (34-36)	**0.001**	.664	**0.001**	**0.019**	0.664
Time to pregnancy across all pregnancies, months	8 (1-24)	24 (15.75-51)	11 (1.25-45)	**0.012**	1.000	**0.009**	0.217	1.000
Time to pregnancy across all pregnancies *(those using ART and fertility-enhancing medication excluded)*	6 (1-18)	18 (9-48)	10 (1-36)	**0.012**	1.000	**0.009**	0.210	1.000
Time to first pregnancy, months	13 (3.5-24)	24 (18-60)	12 (6.4-45)	**0.004**	1.000	**0.003**	0.145	1.000
Time to second pregnancy, months	4.5 (1-16)	18 (15-48)	1 (1-10)	**<0.001**	1.000	**0.001**	**0.002**	1.000
Time to third pregnancy, months	1 (0-19)	24 (24-24)	24 (0-72)	0.058	—	—	—	—
		**N (%)**	**N (%)**	**N (%)**				
Time to first pregnancy categorial, months	<3	6 (22.2)	2 (14.3)	2 (18.9)	.741	—	—	—
	3-6	3 (11.1)	0 (0.0)	1 (7.5)				
	6-12	4 (14.8)	1 (7.1)	2 (13.2)				
	>12	14 (51.9)	11 (78.6)	7 (60.4)				
Time to pregnancy across all pregnancies categorial, months	<3	17 (36.2)	3 (13.6)	6 (30.0)	.061	—	—	—
	3-6	6 (12.8)	0 (0.0)	1 (5.0)				
	6-12	5 (10.6)	1 (4.5)	3 (15.0)				
	>12	19 (40.4)	18 (81.8)	10 (50.0)				
Time to pregnancy across all pregnancies categorial *(those using ART and fertility-enhancing medication excluded), months*								
	<3	11 (44.00)	1 (9.10)	6 (37.50)	**.017**	0.171	**0.045**	0.771
	3-6	5 (20.00)	0 (0.00)	1 (6.30)		0.066	0.177	0.426
	6-12	5 (20.00)	1 (9.10)	1 (2.50)		0.375	0.515	0.701
	>12	4 (16.00)	9 (81.80)	7 (43.80)		**0.001**	**0.001**	0.601

χ²-Test/Fishers exact test. Bold numbers indicate significant group differences. Percentages refer to those women, information was available. Missing information is reported separately.

Abbreviations: IQR, interquartile range; NC, nonclassic; SV, simple virilizing; SW, salt wasting.

^#^Discrepancy due to women using ART after fertility-enhancing medication only at different pregnancies.

### Type of Fertilization, Fertilization Medication, and Invasive Prenatal Diagnostics

More than half (58.6%) of women with NC CAH, 55.6% of women with SV, and 85.7% of women with SW conceived without the use of assisted reproductive technology (ART) or fertility-enhancing medication in any given pregnancy. ART was used by 13.8% of women with NC CAH, 38.9% of women with SV, and 7.1% of women with SW, while fertility-enhancing medication without subsequent ART was used by 27.6% of women with NC CAH, 5.6% of women with SV, and 7.6% of women with SW (*P* = .105). ART was usually only used for the first pregnancy, and in 35.3% of cases in women with SV, significantly more often than in women with NC CAH (11.1%; *P* = .046) or SW (10.1%; *P* = .05). The use of fertility-enhancing medications such as clomiphene or gonadotropins without subsequent ART was reported for 25.7% of pregnancies in women with NC CAH, but only 11.5% in women with SV and 5.0% of women with SW (*P* = .065). Again, the use declined for subsequent pregnancies (Table 2).

### Latency Until Pregnancy

The age of women at their first pregnancy ranged from 19.0 to 47.0 years, with a median age of 30.0 years, and did not significantly differ between phenotypes (*P* = .134).

**Figure 1.**
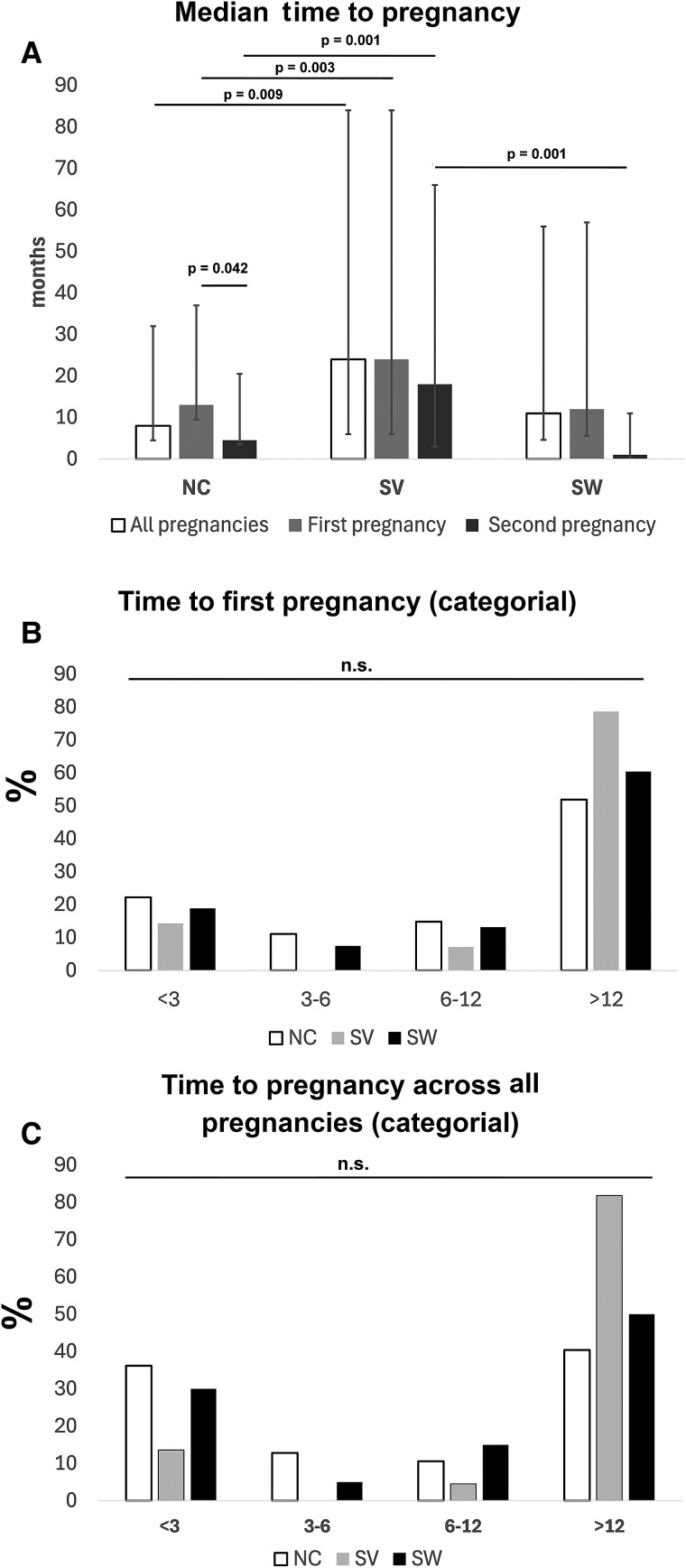
Time to pregnancy varied among groups, with women with NC CAH generally experiencing shorter durations compared to women with SV or SW. Women with SV had significantly longer times to pregnancy overall and for their first pregnancy compared to women with NC CAH (A). The first pregnancy duration for women with NC CAH and SW was similar, with no significant difference between SV and SW. The time to the second pregnancy decreased across all groups, with a significant reduction noted in women with NC CAH. Approximately half of the women with NC and SW required more than 12 months to conceive their first child (B), and this proportion remained consistent across all pregnancies (C). For women with SV, the delay was even more pronounced, with 78.6% taking longer than 12 months to become pregnant, a figure that increased to 81.8% across all pregnancies (n.s.) Kruskal-Wallis-Test with post hoc Dunn-Bonferroni-Test and Wilcoxon signed-rank test. Abbreviations: NC, nonclassic; SV, simple virilizing; SW, salt wasting.

Across all pregnancies, the median time to pregnancy was 8 months (interquartile range [IQR] 1-24) for women with NC CAH, 24 months (IQR 15.75-51.0) for women with SV, and 11 months (IQR 1.25-45) for women with SW. The time to pregnancy was significantly longer for women with SV in comparison to women with NC CAH (*P* = .009). Similarly, for the first pregnancy, it took women with NC CAH 13 months (IQR 3.5-24) to become pregnant, which was significantly shorter than for women with SV (24 months, IQR 18-60; *P* = .003). In women with SW, the median time to first pregnancy was 12 months (IQR 6.4-45 months; *P* = 1.000) (SV vs SW *P* = .145) (Table 2, Fig. 1B). As only a minority of women had 3 or more children, we only explored if there was a difference in time to pregnancy between the first and second pregnancy.

For the second pregnancy, time decreased in all groups, but the decrease was only significant in women with NC CAH to 4.5 months (IQR 1-16) (*P* = .042). Time to pregnancy was still longer for women with SV than with NC CAH when excluding all patients having received pregnancy-enhancing medication or ART (Fig. 1A, Table 2).

In detail, approximately half of the women with NC and SW required more than 12 months to conceive their first child, and this proportion remained consistent across all pregnancies. For women with SV, the delay was even more pronounced, with 78.6% taking longer than 12 months to become pregnant, a figure that increased to 81.8% across all pregnancies (n.s.) (Figure 1C, Table 2).

### Duration of Pregnancy

**Figure 2.**
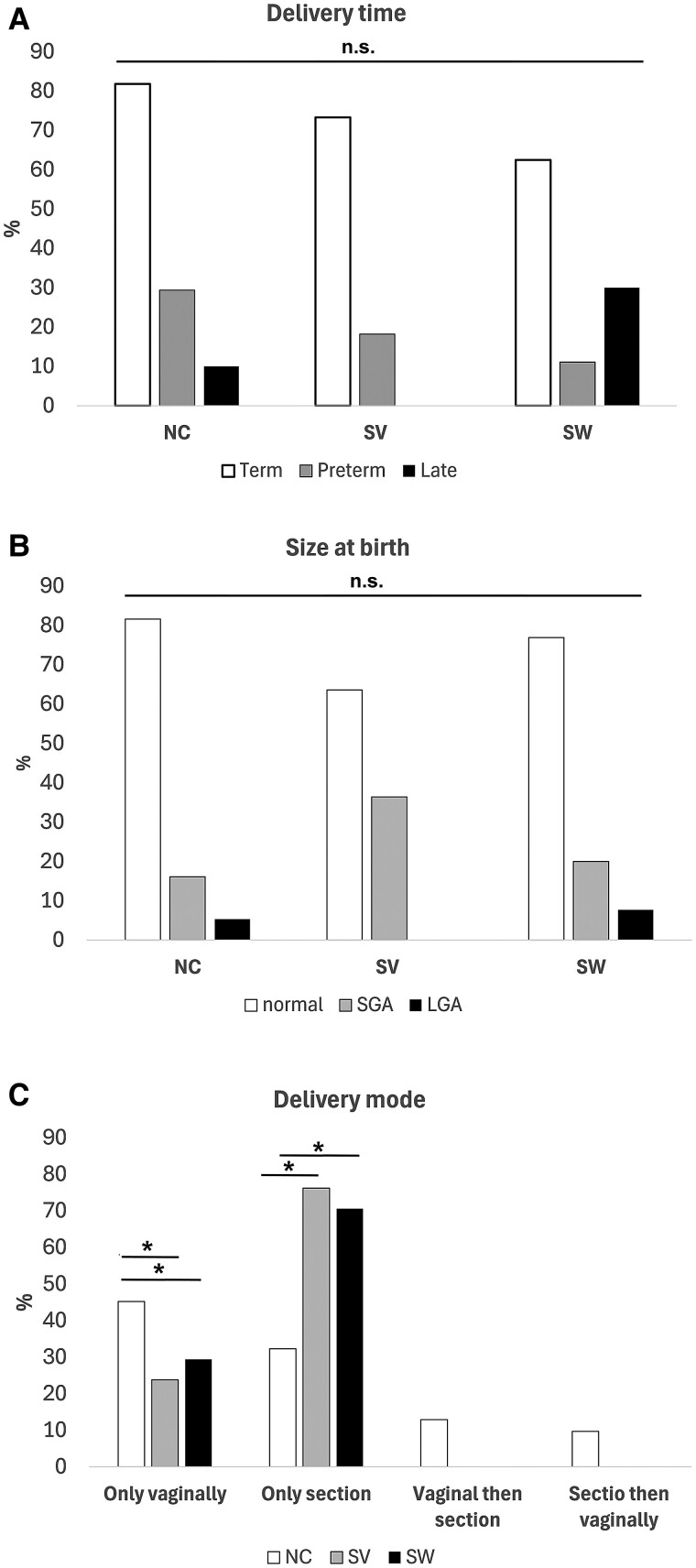
Term birth rates did not significantly differ between groups (A). Preterm births were reported for 17 children across the cohort. Approximately one-third of the children born to women with SV were small for gestational age (SGA), which was higher compared to 16.1% in the NC CAH group and 20.0% in the SW group on a trend level (*P* = 0.088) (B). Only 3 children were reported as large for gestational age (LGA). In 60.4% of pregnancies among women with NC CAH, children were born vaginally, compared to 21.4% in women with SV and 34.6% in women with SW (*P* = .002). Across all pregnancies, cesarean section was the only mode of delivery in 32.3% of cases for women with NC CAH, compared to 76.2% for women with SV and 70.6% for women with SW (C). Abbreviations: NC, nonclassic; SV, simple virilizing; SW, salt wasting.

In women with NC CAH, 81.8% of children were born at term, compared to 73.3% in women with SV and 62.5% in women with SW (*P* = .266). Seventeen children in the entire cohort were born preterm, but only 4 were born late (n.s.) (Fig. 2A). About a third of the children in women with SV, for whom sufficient information was available, were born SGA (36.4%) compared to 16.1% in the NC CAH group and 20.0% in the SW group (*P* = .088) (Fig. 2B). Only 3 children in total were born LGA.

Birthweight was not affected by HCeq or type of GC used during pregnancy in a multivariate model adjusted for phenotype, birthweight, week of birth, and sex (Supplement Table S3 [13]).

### Mode of Delivery

In 60.4% of pregnancies among women with NC CAH, children were born vaginally, compared to 21.4% in women with SV and 34.6% in women with SW (*P* = .002). Across all pregnancies, cesarean delivery was the only mode of birth in 32.3% of cases for women with NC CAH, compared to 76.2% for women with SV and 70.6% for women with SW (Fig. 2C). Of the 42 women who had a cesarean delivery for their first birth, 10 were unplanned, including 4 emergency cesarean deliveries (Supplement Table S4 [14]). These emergency procedures were mainly due to pathological cardiotocograms (CTGs). For those who had subsequent cesarean deliveries, labor weakness and obstructed labor were the primary reasons for opting out of vaginal delivery. Additionally, of the patients who delivered vaginally at their first birth, 11 (40.7%) reported the need for an assistive device such as suction cup, forceps, or episiotomy. Patients with SV and SW were particularly affected, compared to 33.3% in the NC CAH group (n.s.) (Supplement Table S4 [13]). A significant proportion of women who underwent genitourinary surgery had delivered only vaginally (25% of women with SW and 14.3% of women with SV). The one woman with a 0 mutation in the SW group had given birth to 2 children, both of whom were born via cesarean delivery.

### CAH-Specific Treatment Before Pregnancy

Among women with NC CAH, 63.3% reported regular GC intake before their first pregnancy, compared to 94.4% of women with SV and 92.9% of women with SW (*P* = .079). The median GC equivalent dose before the onset of pregnancy was 12.0 mg/day (IQR 8-20) in women with NC CAH, 16.5 mg/day (IQR 20-30) in women with SV, and 30 mg/day (IQR 25-32.5) in women with SW (*P* < .001). All women from the SW group and 2 women from the SV group regularly took mineralocorticoids before pregnancy (Table 3).

**Table 3.** Glucocorticoid management

	NC (N = 34)	SV (N = 21)	SW (N = 17)	*P*	NC vs SW	NC vs SV	SV vs SW
Rate of regular GC medication before first pregnancy (%)	19 (63.3)	17 (94.4)	17 (92.9)	.079	0.109	0.064	0.658
None	10 (36.7)	2 (5.6)	1 (7.1)				
Missing	2 (5.9)	3 (14.3)	2 (11.8)				
Type of GC							
HC	10 (45.5)	3 (20.0)	9 (64.3)	**.05** *^a^*	0.270	0.111	**0.016**
Pred	9 (40.9)	9 (60.0)	4 (28.6)				
Dex	3 (13.6)	2 (13.3)	0 (0.0)				
Pred + HC	0 (0.0)	1 (6.7)	1 (7.1)				
Missing	2	3	2				
MC intake	0 (0)	2 (10.5)	15 (100)				

	Median (IQR)	Median (IQR)	Median (IQR)		NC vs SW	NC vs SV	SV vs SW
HCeq before first pregnancy (mg)	12 (8-20)	28 (20-30)	30 (25-32.5)	**<.001**	**<0.001**	**<0.001**	1.000
GC intake during first pregnancy							
Never	8 (27.6)	0 (0.0)	0 (0.0)				
New	2 (6.9)	1 (9.1)	0 (0.0)				
Same	9 (31.0)	5 (45.5)	4 (33.3)				
Increase	7 (24.1)	3 (27.3)	6 (50)				
Decrease	1 (3.4)	2 (18.2)	2 (16.7)				
Terminated	2 (6.9)	0 (0.0)	0 (0.0)				
Change of GC type	2 (11.8)	2 (16.7)	3 (25)				
Missing	3 (8.8)	10 (48.6)	5 (29.4)				
HCeq first pregnancy (mg)	16.5 (10-30)	30 (21.3-38.1)	35 (25-40)	**<.001**	**<0.001**	**<0.001**	1.000
HCeq second pregnancy (mg)	15 (10-30)	20 (15-30)	25 (20-40)	**.006**	**0.004**	0.218	0.457
HCeq third pregnancy (mg)	15 (15-30)	17.5 (15-20)	30 (25-40)	**.003**	**0.015**	1.000	**0.010**

χ²-test/Fisher exact test, Kruskal-Wallis-Test with post hoc Dunn-Bonferroni test. Bold numbers indicate significant group differences.

Abbreviations: Dex, dexamethasone; GC, glucocorticoid; HC, hydrocortisone; HCeq, hydrocortisone equivalent dose of glucocorticoid; MC, mineralocorticoid; NC, nonclassic; Pred, prednisone/prednisolone; SV, simple virilizing; SW, salt wasting.

^*a*^HC vs synthetic GC.

### CAH-Specific Treatment During Pregnancy

Almost all women with classic CAH took GC regularly during pregnancy, whereas this was only the case for 63.3% in the NC group (*P* = .004). The calculated hydrocortisone equivalent (HCeq) during the first pregnancy increased to 16.5 mg (IQR 10-30) in women with NC CAH (*P* = .018) but remained stable in women with SV (30 mg, IQR 21.3-38.1; *P* = .684) and SW (35 mg; IQR 25-40; *P* = .091). In 7 women, the type of GC changed compared to before pregnancy, resulting in a calculated increase in HCeq dosage in 3 and a decrease in dosage in another 3 women. Mineralocorticoid preparations were taken without change during pregnancy by all patients in the SW group and by 2 patients in the SV group. For the second pregnancy, HCeq did not significantly change in any group compared to the first pregnancy (data not shown). Information on CAH-specific treatment during pregnancy can be found in Table 3.

### Complications During Pregnancy

Pregnancy complications were equally common across all 3 phenotypes. Among all groups, cervical insufficiency was one of the most frequently reported complications, occurring 7 times in just the first pregnancies. In total, 2 women in the NC CAH group (5.9%) and 1 woman each in those with SV (4.8%) and SW CAH (5.9%) developed preeclampsia or gestational hypertension (*P* = .982). Gestational diabetes was more common in women with SV (19%) compared to women with NC CAH (2.9%) and SW (.0%) (*P* = .05). A detailed overview of the complications during the initial pregnancy and delivery can be found in Supplement Table S3 [13].

### Condition of Children After Birth

In each group one child presented with pathological Apgar scores after birth (*P* = .682).

### Desire to Have Further Children

Of the 39 women (17 NC: 58.6%, 12 SV: 68.4%, 9 SW: 60.0%; *P* = .780) in our cohort who indicated they did not wish to have more children, 8 (21.1%) explicitly cited their CAH diagnosis as the reason (5 NC, 1 SV, 2 SW; *P* = .389). In the NC group, the time to first pregnancy was longer in those stating that they did not want to have further children (*P* = .047).

## Discussion

Our study sheds light on the fertility and pregnancy outcomes of patients with CAH. Previous studies have shown low fertility rates in CAH, but the general perception today is that those who wish to conceive, do conceive and pregnancy rates are equal to the general population [10, 23, 24]. This is, however, the first study to show that the latency to pregnancy is prolonged in the majority of patients with CAH irrespective of the phenotype and ART or fertility-enhancing medication frequently have to be used in a high percentage to achieve pregnancy.

Our findings however did not aim to compare overall fertility rates across the 3 CAH phenotypes, the data indicates consistent pregnancy outcomes, including live births and miscarriages, for those who did conceive.

An extensive epidemiological study from Sweden, targeting a similar age group, reported a lower likelihood of women with CAH having at least one child (25.4%) compared to the control population (45.8%) [10]. In line with the results of our study, no marked difference in the number of children existed among those who had given birth, although a smaller proportion of women with the SW phenotype became mothers compared to those with SV or NC CAH. This indicates that a more severe phenotype does not necessarily result in less favorable pregnancy outcomes. However, among those women for whom genotype data were available, there was only one woman with a null genotype in our cohort who had given birth to a child. In line with this, successful pregnancies in such women have rarely been reported in the literature [12].

Multiple factors influence the reported lower fertility rate in women with CAH. One such factor is the decreased inclination toward motherhood. Casteras et al [25] found that only 23.6% of 106 women diagnosed with classic CAH expressed a desire for pregnancy, whereas Bidet et al [26] reported that half of the 190 women with NC CAH harbored such aspirations. These patterns might be influenced by several social and medical reasons. For instance, compared to healthy counterparts, fewer female patients with CAH are in heterosexual relationships, with a pronounced prevalence of bisexuality or homosexuality among them [27]. As we present the results of a preselected cohort based on motherhood, we cannot comment on the sexual orientation or relationship status for women with CAH in general.

An additional factor may be the results of genital reconstructive surgeries, as these procedures can sometimes be unsatisfactory, leading to patient discontent with their genital appearance and function [28].

Interestingly, many women in our study who had children did not wish for more due to their CAH diagnosis. This sentiment was prevalent among women with NC CAH. This reluctance can therefore not merely be attributed to genital abnormalities, especially considering that the time taken to become pregnant for women with SV was almost 2 years, while it was significantly shorter for those with SW and NC CAH. Nonetheless, time to first pregnancy was longer in those women stating no further desire to have children. Regarding fertility interventions, in line with the longer time to become pregnant, the use of any kind of fertility treatment was considerable high in our cohort. ART was significantly higher in women with SV than in women with a SW or NC phenotype, while fertility-enhancing medication tended to be more commonly used in women with NC CAH.

Previous studies have shown that, overall, women with SW have fewer children than those with the SV or NC CAH phenotype. As mentioned before, in our cohort, only one woman with a null phenotype had given birth. This may suggest that, among women with SW, those with some residual enzymatic activity were overrepresented in our preselected cohort and as our study only included women who had either given birth or at least experienced pregnancy, the picture may be skewed, potentially excluding women who either never attempted to conceive or gave up after unsuccessful attempt.

We also observed a prolonged duration from the start of attempts to conceive to successful conception, between 1 and 2 years, which is longer than reported elsewhere [29]. Even when a pregnancy is desired and sexual intercourse is feasible, the inherent hormonal imbalance in women with CAH might lead to anovulatory or irregular cycles due to adrenal steroid production. Furthermore, elevated progesterone levels could directly inhibit conception at the endometrial level [2]. GC use in women with NC CAH has been associated with a shorter time to conceive, which in turn has been associated with lowered androgen levels preconceptionally [29]. In our cohort, 69% of women with NC CAH were using GCs preconceptionally and the majority was continuing treatment throughout pregnancy. Time to pregnancy significantly decreased in women with NC CAH for subsequent pregnancies without further dosage adjustments, indicating that the learning experience during the first pregnancy might have helped to accelerate successful conception in following pregnancies.

It is unclear why 5 women were taking dexamethasone throughput pregnancy, as this did not seem to be in the context of prenatal treatment of classic CAH. Dexamethasone treatment is usually not recommended [2, 30], as it can surpass the placenta barrier and potentially lead to adverse outcomes in child and mother [30, 31]. However, it does not necessarily correlate with an increased incidence of SGA births [2, 32]. In line, birthweight was not affected by HCeq or type of GC in our study.

The overall rate of SGA in our cohort stood at around 20%. Similarly, in a previous study, 16% of children were reported to be born SGA [26]. In contrast, the Swedish cohort showed no SGA births in the SW and NC groups, while it was 9.3% in women with the SV phenotype, which was notably double the rate observed in the control group [12]. It is important to note that, in contrast to our study, the Swedish cohort defined SGA as having a birth weight more than 2 SD below the sex-specific mean for gestational age. This definition includes only about 2.4% of the general population as SGA. Therefore, the prevalence of SGA may not be directly comparable across different studies in the literature.

In has been reported that GC use in women with NC CAH not only helps them to successfully conceive but also that it is associated with a decreased rate of spontaneous miscarriages [12, 29]. The rate of spontaneous abortions across all groups in our study was 18.0% with no group differences and rates were generally comparable to earlier studies [26, 33, 34]. Whether GC should be continued in the course of pregnancy in NC CAH is, however, a matter of debate as the lower rate of miscarriages is not a consistent finding [34]. All women in our cohort who had spontaneous abortion during their first pregnancy were taking a GC.

GC dosage was not increased in all women during pregnancy as recommended [35] and the increase of median 4 mg in HCeq was only significant in women with NC CAH. This may be due to increased GC prior to pregnancy to lower the adrenal steroid precursors, including progesterone, to increase the chances of conceiving.

Our study also touched upon pregnancy complications, with only a few women reporting gestational diabetes, hypertension, or preeclampsia. This translates to a rate of gestational diabetes of 8.3% across the whole group. Although the total numbers were small, gestational diabetes seemed to be more prevalent in the SV group in our cohort. Hirschberg et al reported on an increased rate of gestational diabetes 4.9% vs 1.4% for the control group [12]. While a systematic review on this topic found a comparable absolute risk for gestational diabetes of 7.3% [23] to what is expected for all pregnancies in the general population [36]. Discrepancies in these “metabolic” complications between studies may depend on the basal characteristics of the cohort and differences in GC treatment regimens throughout pregnancies.

In addition, women with CAH have a higher rate of obesity and may subsequently already have a higher rate of subclinical insulin resistance even before pregnancy [37], factors that may predispose them to gestational diabetes.

For Germany, where most of the included patients came from, the rate of gestational diabetes has been reported to lie between 4.6% to 6.8% for a comparable time period [38]. The relatively high glucocorticoid dose necessary to conceive and given during pregnancy may explain the elevated rate of gestational diabetes. For preeclampsia, our prevalence is in accordance with the literature [12, 23].

We observed a notable trend of high planned cesarean delivery rates across all CAH groups. Women with classic CAH often have a more constricted pelvic anatomy, often leading to recommendations for elective cesarean delivery. The rate of 70.6% for women with SW was lower than the reported 91% reported elsewhere [12]. The fact that general perinatal complications, and in particular emergency cesarean deliveries, were not increased, despite these women having undergone an average of 2 genital surgeries, suggests that cesarean delivery in these women is not obligatory. Decisions should therefore be made considering all risk factors and not based on the CAH diagnosis alone.

The reasons for the significant number of women with NC CAH who choose cesarean deliveries remains unclear. As a majority of patients with nonclassic CAH had GC replacement doses to conceive, uncertainty about stress dosing during longer periods of labor in vaginal delivery compared to cesarean section might contribute to this fact. Notably, while the percentages of scheduled cesarean deliveries vary widely across medical centers and nations, they seldom surpass one-third of all childbirths [39-41]. In earlier studies, section rates in women with NC CAH were ranging from 28.7% to 33% [30]. Studies have shown that cesarean sections without medical indications can lead to adverse outcomes [41]. This suggests that there might be a gap in understanding and highlights the need for better education and awareness among healthcare providers.

## Strengths and Limitations

A strength of our study is the multicenter character and the detailed documentation of pregnancy courses. A limitation of this study is its retrospective design and the use of questionnaires that may be susceptible to recall bias and missing data. In addition, rates of pregnancy complications were compared to the general population, and we did not include a matched control group in our study. Given the multicentric nature of the study spanning different countries and corresponding healthcare systems, designing an adequately matched control group was challenging.

We also recognize the challenges in determining the optimal period for conception in patients undergoing assisted reproductive techniques. The decision on the timing of treatment often depends on the individual choices of the patients and their partners, rather than the underlying medical condition. Nevertheless, for women under the age of 35 attempting their first pregnancy, ART is typically recommended or pursued only after 1 year of unsuccessful attempts at conceiving through regular, unprotected intercourse [42]. This highlights the ongoing difficulties in achieving pregnancy for women with CAH.

As the composition of the study population may be special, the results may not be necessarily translated to the general population of women with CAH.

## Conclusion

Our study suggests that despite a longer latency to the first pregnancy for women with CAH, the spontaneous abortion rates or pregnancy complications are not increased. The high rate of cesarean sections in women with NC CAH demand further investigation. The fact that general perinatal complications and in particular emergency cesarean sections were not increased despite these women having undergone an average of 2 genital surgeries suggests that cesarean section in these women is not obligatory. Decisions should therefore be made considering all risk factors and not based on the CAH diagnosis alone.

## Data Availability

The datasets analyzed for the current study are available from the corresponding author upon reasonable request.

## Abbreviations

21OH: 21-alpha-hydroxylase
ART: assisted reproductive technology
CAH: congenital adrenal hyperplasia
GC: glucocorticoid
HCeq: hydrocortisone equivalent doses of glucocorticoid
IQR: interquartile range
LGA: Large for gestational age
NC: nonclassic
n.s.: not significant
SGA: small for gestational age
SV: simple virilizing
SW: salt wasting
